# ABE8e adenine base editor precisely and efficiently corrects a recurrent *COL7A1* nonsense mutation

**DOI:** 10.1038/s41598-022-24184-8

**Published:** 2022-11-16

**Authors:** Adam Sheriff, Ina Guri, Paulina Zebrowska, Virginia Llopis-Hernandez, Imogen R. Brooks, Stavroula Tekkela, Kavita Subramaniam, Ruta Gebrezgabher, Gaetano Naso, Anastasia Petrova, Katarzyna Balon, Alexandros Onoufriadis, Dorota Kujawa, Martyna Kotulska, Gregory Newby, Łukasz Łaczmański, David R. Liu, John A. McGrath, Joanna Jacków

**Affiliations:** 1 St John’s Institute of Dermatology, Faculty of Life Sciences and Medicine, King’s College London, 9th Floor Tower Wing, Guy’s Hospital, Great Maze Pond Road, London, SE1 9RT UK; 2 Hirszfeld Institute of Immunology and Experimental Therapy, Polish Academy of Sciences, Wroclaw, Poland; 3 Molecular and Cellular Immunology Unit, UCL GOS Institute of Child Health, London, UK; 4 Merkin Institute of Transformative Technologies in Healthcare, Broad Institute of MIT and Harvard, Cambridge, MA USA; 5 Department of Chemistry and Chemical Biology, Harvard University, Cambridge, MA USA; 6 Howard Hughes Medical Institute, Harvard University, Cambridge, MA USA

**Keywords:** Genetics, Molecular biology, Medical research, Molecular medicine

## Abstract

Base editing introduces precise single-nucleotide edits in genomic DNA and has the potential to treat genetic diseases such as the blistering skin disease recessive dystrophic epidermolysis bullosa (RDEB), which is characterized by mutations in the *COL7A1* gene and type VII collagen (C7) deficiency. Adenine base editors (ABEs) convert A-T base pairs to G-C base pairs without requiring double-stranded DNA breaks or donor DNA templates. Here, we use ABE8e, a recently evolved ABE, to correct primary RDEB patient fibroblasts harboring the recurrent RDEB nonsense mutation c.5047 C > T (p.Arg1683Ter) in exon 54 of *COL7A1* and use a next generation sequencing workflow to interrogate post-treatment outcomes. Electroporation of ABE8e mRNA into a bulk population of RDEB patient fibroblasts resulted in remarkably efficient (94.6%) correction of the pathogenic allele, restoring *COL7A1* mRNA and expression of C7 protein in western blots and in 3D skin constructs. Off-target DNA analysis did not detect off-target editing in treated patient-derived fibroblasts and there was no detectable increase in A-to-I changes in the RNA. Taken together, we have established a highly efficient pipeline for gene correction in primary fibroblasts with a favorable safety profile. This work lays a foundation for developing therapies for RDEB patients using ex vivo or in vivo base editing strategies.

## Introduction

Dystrophic epidermolysis bullosa (DEB) is a severe inherited disorder characterized by skin blistering and epithelial fragility^1^. DEB is caused by mutations in the *COL7A1* gene that encodes type VII collagen (C7)^2^, the main constituent of anchoring fibrils (AFs) which tether the epidermis to the underlying dermis^3^. *COL7A1* mutations disrupt the synthesis, secretion, or processing of C7, resulting in structurally defective AFs and reduced dermal-epidermal cohesion, which leads to blistering and poorly healing chronic wounds, often complicated by scarring. Recessive Dystrophic Epidermolysis Bullosa (RDEB), which is inherited in an autosomal recessive fashion, is the most severe form of DEB. Therapies which reverse the effects of causative *COL7A1* mutations could therefore resolve a pressing unmet need for RDEB patients.

At present there are no curative treatments for RDEB^4^. Despite several early phase clinical trials involving gene, cell, protein, and small molecule therapies, nearly all current therapies only aim to alleviate symptoms, with wound care remaining the cornerstone of treatment^5^. Most gene therapy approaches in RDEB have focused on the addition of full-length *COL7A1* cDNA into cells and then transplanting or injecting *COL7A1*-supplemented autologous keratinocytes (KCs), fibroblasts (FBs) or skin equivalents^4^. Most recently, an early phase clinical trial involving a topical herpes simplex virus-1 vector with a *COL7A1* cargo promoted wound healing in patients with RDEB^6^.

In contrast, gene editing approaches aim to permanently correct pathogenic mutations at the genomic level and thereby mitigate some of the drawbacks of gene addition therapy such as insertional mutagenesis, inaccurate spatial–temporal gene expression and the progressive extinction of the transgene which may necessitate repeated therapy^7–9^. Recent advances in genome editing technologies now enable the installation of desired DNA changes with higher efficiencies and without double-stranded DNA breaks (DSBs), which cause uncontrolled insertion or deletion mutations at the break site and numerous undesired cellular consequences of DSBs such as chromosomal abnormalities and p53 activation^8,10^. Base editors (BEs) have the potential to reverse the four most common single-base substitutions at precise targets in the genome. Of note, BEs do not institute DSBs and, unlike CRISPR-Cas9, do not rely on homology-directed repair (HDR), therefore potentially offering greater correction efficiency in slowly dividing or post-mitotic cells such as skin FBs and KCs with fewer by-products^11,12^.

Specifically, adenine base editors (ABEs) install A-T to G-C base pair changes within a confined editing window defined by a programmable single guide RNA (sgRNA)^13^. ABE is especially relevant to RDEB in which approximately 75% of pathogenic mutations are point mutations, half of which are targetable by ABEs^14^*.* ABEs are suited to reverse premature termination codon (PTC) mutations, caused by C-to-T changes, that promote nonsense-mediated mRNA decay^15^ and a complete absence of C7 expression, causing the most severe disease phenotypes. ABE has been shown in a previous study to correct 23.8% and 8.2% of mutated alleles in a population of RDEB fibroblasts^16^, however the clinical translation of gene editing demands higher efficiencies to obviate selection markers for corrected cells, restore AF and facilitate efficacious ‘*in-vivo’* gene editing therapy directly on patients’ skin. To increase the efficiency and specificity of ABE, new versions such as ABE7.10 and ABE8e have since been developed through directed evolution of the TadA domain^13,17,18^.

Here, we report ABE8e-mediated correction of the *COL7A1* c.5047 C > T (p.Arg1683Ter) mutation in exon 54 in fibroblasts derived from a patient with RDEB. We demonstrate efficient genomic DNA correction of the mutated allele (94.6%) which restores *COL7A1* mRNA and C7 protein expression with no detectable off-target effects at predicted sites at the DNA level or increase in A-to-I changes on the transcriptome. When delivered into RDEB fibroblasts by electroporation of therapeutically relevant doses of ABE8e mRNA and a single guide (sgRNA), a silent bystander mutation installed within the editing window by ABE8e was diminished whilst maintaining efficient reversal of the c.5047 C > T mutation. This study uncovered a potential therapeutic strategy for RDEB which directly corrects the causative *COL7A1* mutation in primary patient-derived fibroblasts with an efficient correction rate and a favourable safety profile. This work paves the way for therapies for RDEB patients using ex vivo or in vivo base editing strategies.

## Results

### Patient selection and *COL7A1* mutation analysis

**Figure 1:**
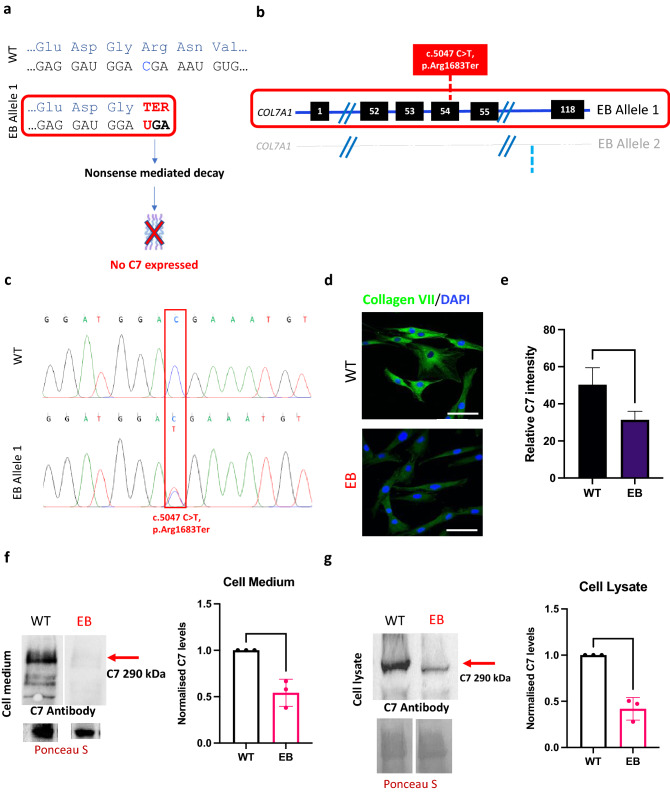
Compound heterozygous mutations in patient (EB) fibroblasts lead to reduced C7 expression. (**a**) c.5047 C > T on allele 1 is a nonsense mutation which changes Arginine to a stop codon, leading to a truncated mRNA transcript which is degraded by nonsense-mediated decay. This typically leads to no C7 produced from the affected allele and the blistering phenotype. (**b**) Schematic of the 2 alleles of the *COL7A1* gene in patient EB, illustrating compound heterozygosity. The c.5047 C > T mutation on allele 1 is in Exon 54 and is the mutation of interest. The c.7344 + 1 G > A mutation on allele 2 is in Intron 95 of the gene. (**c**) Sanger sequencing of DNA isolated from EB fibroblasts confirms the nonsense mutation in Exon 54 in comparison to wild-type sequences from Wild Type fibroblast (WT) controls. (**d**) Immunostaining for C7 reveals markedly reduced expression in EB fibroblasts compared to normal. Representative images taken at 40× magnification shown. Scale bar is 20 µm. (**e**) Quantification of relative immunofluorescence (IF) intensity of C7 demonstrates a significant reduction in C7 in EB cells compared to WT. t-test performed, *p* value < 0.0001. Western Blot analysis found EB fibroblasts secrete approximately 50% of normal levels of full length C7 into cell medium (**f**) and produce 50% of normally expressed full-length C7 in the cell lysate (**g**). Total protein loading was controlled using Ponceau S for cell lysate and cell medium. 3 technical replicates and t test performed showing significance (*p* value < 0.05). Full blots are presented in Supplementary Fig. S1b–e.

The patient fibroblasts used for this study were selected from a comprehensive database of RDEB patients clinically examined and diagnosed at St John’s Institute of Dermatology (London, UK). We screened patients for premature termination codon (PTC) mutations in *COL7A1* that were targetable by ABE by analyzing the region surrounding each mutation on a reference genome for an appropriately placed ‘NGG’ protospacer adjacent motif (PAM) site. Of the 31 RDEB point mutations in *COL7A1* screened, 16 (52%) were targetable by base editing, of which 7 (23%) were PTC mutations. For this study, we selected the c.5047 C > T mutation which converts an arginine at position 1683 into a stop codon (p.Arg1683Ter), as an example of a PTC mutation which engenders a truncated mRNA transcript and no C7 protein production (Fig. 1a). The c.5047 C > T mutation is recurrent amongst RDEB patients worldwide and was present in 5 patients recruited for an early phase gene therapy clinical trial^6^, demonstrating the global relevance of targeting this mutation and the opportunity to recruit participants for future clinical study. For the current study, fibroblasts were isolated from a patient who was a compound heterozygote for the c.5047 C > T (p.Arg1683Ter) and c.7344 + 1 G > A (IVS95 + 1) mutations in *COL7A1* (Fig. 1b). Fibroblasts are relevant in RDEB as they are the major secretors of C7, which is also generated by KCs. DNA was extracted from the RDEB patient fibroblasts (EB) and wild type fibroblasts (WT) and primers were used to amplify fragments of both exon 54 and intron 95 around the known mutations in *COL7A1*. Sanger sequencing found overlapping peaks in the chromatograms from patient cells compared to WT which confirmed a nonsense mutation at the specific loci c.5047 C > T (Fig. 1c). The impact of the mutations on C7 expression was evaluated by fluorescence microscopy and western blot. Fluorescence microscopy using LH7.2 antibody, which recognises the carboxy terminal of the C7 dimer, indicated that C7 was present in both cell lines, however, the production of C7 was markedly diminished in EB fibroblasts compared to WT (Fig. 1d). Quantification of relative C7 intensity from immunofluorescence and analysis confirmed significantly reduced C7 expression in EB fibroblasts compared to WT (Fig. 1e). Similarly, western blot analysis of C7 showed a band at ~ 290 kDa (full-length C7) in both the cell lysates and supernatant of EB fibroblasts and WT, with comparatively less C7 in the patient (Fig. 1f,g). Normalized densitometry revealed that EB fibroblasts secreted around 60% of the C7 secreted by WT into the cell medium (Fig. 1f) and expressed approximately 50% of the C7 expressed by WT from the cell lysates (Fig. 1g). Although the PTC mutation on allele 1 leads to no C7 expression, residual C7 expression is maintained by the gene product from allele 2 which contains a donor splice site mutation, c.7344 + 1 G > A. Mutations which occur at the splice site, the boundary of an exon and an intron, can disrupt RNA splicing resulting in the loss of exons or the inclusion of introns and an altered protein-coding sequence. The c.7344 + 1 G > A mutation results in in-frame exon skipping which was confirmed using RT-PCR demonstrating EB fibroblasts had transcripts of a shorter length in the Exon/Intron 95 region (Supplementary Fig. S1a).

### ABE8e fully corrects the *COL7A1* truncation in RDEB patient fibroblasts

**Figure 2:**
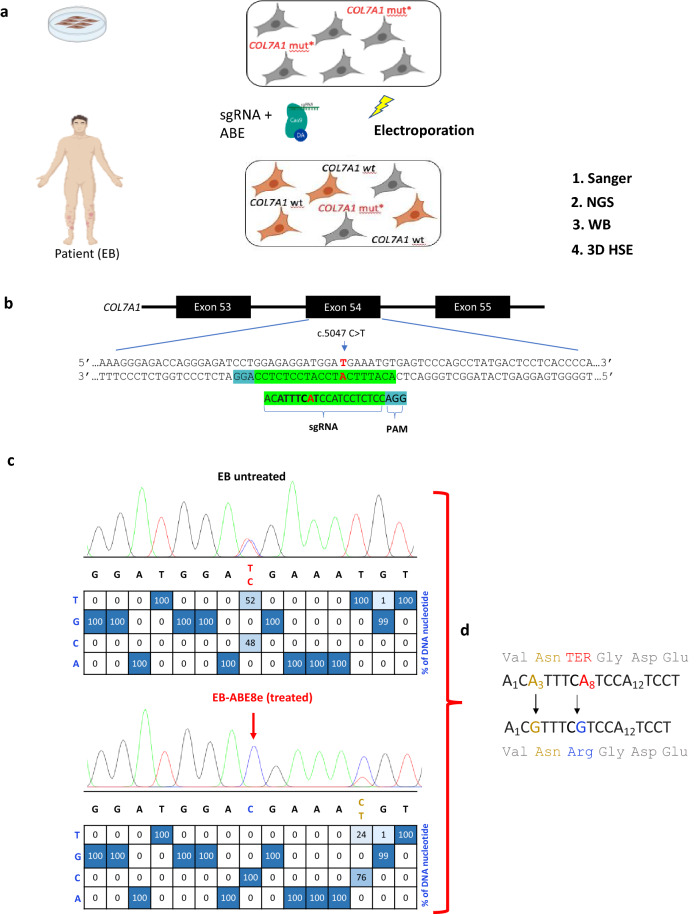
Design of base editing experiments and initial outcomes. (**a**) Strategy to correct a bulk population of patient-derived fibroblasts by electroporating ABE with a single guide (sg)RNA. Post-editing analysis used Sanger sequencing and Next generation sequencing (NGS) on both the DNA and RNA levels. Studies on C7 protein involved Western blotting (WB) and 3-Dimensional human skin equivalents (3D HSE) generated from fibroblasts and keratinocytes. (**b**) The chemically modified sgRNA used for all base editing experiments was designed with the mutated nucleotide in position 8 (shown in red) of its 20-base protospacer, on the complementary strand. The editing window (positions 3–9) is in bold and the required ‘NGG’ protospacer adjacent motif (PAM) site is highlighted in blue. (**c**) *In-silico* analysis of Sanger sequencing data suggested all alleles containing the mutation (shown in red) were corrected. Sequencing also uncovered a bystander mutation had been introduced in 76% of alleles (**d**) Correction of the nonsense mutation in position 8 reversed the pathogenic amino acid change from a termination codon (red) to arginine (blue). The bystander mutation, introduced at position 3, conferred a silent asparagine-to-asparagine amino acid change (gold). No other nucleotides in the protospacer were targeted.

A bulk population of EB patient fibroblasts was electroporated with 5 µg sgRNA together with either 2 µg mRNA of ABE8e or ABE7.10, a previous-generation ABE version. Editing outcomes were interrogated using Sanger sequencing and high-throughput sequencing with Illumina’s Next Generation Sequencing (NGS) (Fig. 2a). The sgRNA, compatible with both versions of ABE, is a single-stranded 20 nucleotide stretch of bases complementary to the genome proximal to the mutation. This enabled the design of a sgRNA which could be deployed to guide the ABE to the target site of the c.5047 C > T mutation. ABE enzymatically converts adenine bases to guanine, therefore the sgRNA targeted the complementary strand to reverse this mutation at position c.5047. The sgRNA was designed using the BE-designer online web tool^19^ to optimally position the protospacer so that the target nucleotide is within the editing window and appropriately spaced from the NGG PAM required by SpCas9 (Fig. 2b). The c.7344 + 1 G > A mutation had no available NGG PAM to mediate editing with wild type SpCas9. EditR^20^ was used for in silico analysis of base editing efficiency by calculating the composition of individual nucleotides within the sgRNA-targeted sequence. The editing efficiency of the base editor (percentage of C > T conversions) was calculated using the following equation: 50% is the baseline T nucleotide frequency at the mutation locus in EB fibroblasts.

$$editing\, efficiency \left( \% \right) = \frac{50 - T\, frequency\, in\, edited\, cells}{{50}} \times 100$$

A complete reversal in the peak at position c.5047 was visualized in the ABE8e edited (EB-ABE8e) bulk fibroblast line showing a genomic change at the target locus. Before editing, the proportion of T bases was 52% and the proportion of C nucleotides was 48%. EditR illustrates that after editing with ABE8e, the proportion of the C allele became 100% and the mutated T allele was reduced to 0%. This initial analysis estimates an editing efficiency of 100%, meaning that the mutation was reversed in almost every cell in the bulk population. A shift in the peak at position 3 of the editing window was also observed, suggesting a bystander mutation had occurred (Fig. 2c). Correction of the nonsense mutation in position 8 reversed the pathogenic amino acid change and the bystander mutation, introduced at position 3, conferred a silent asparagine-to-asparagine amino acid change (Fig. 2d).

### Off-target DNA and RNA editing analysis on base edited RDEB fibroblasts showed a favourable safety profile

**Figure 3:**
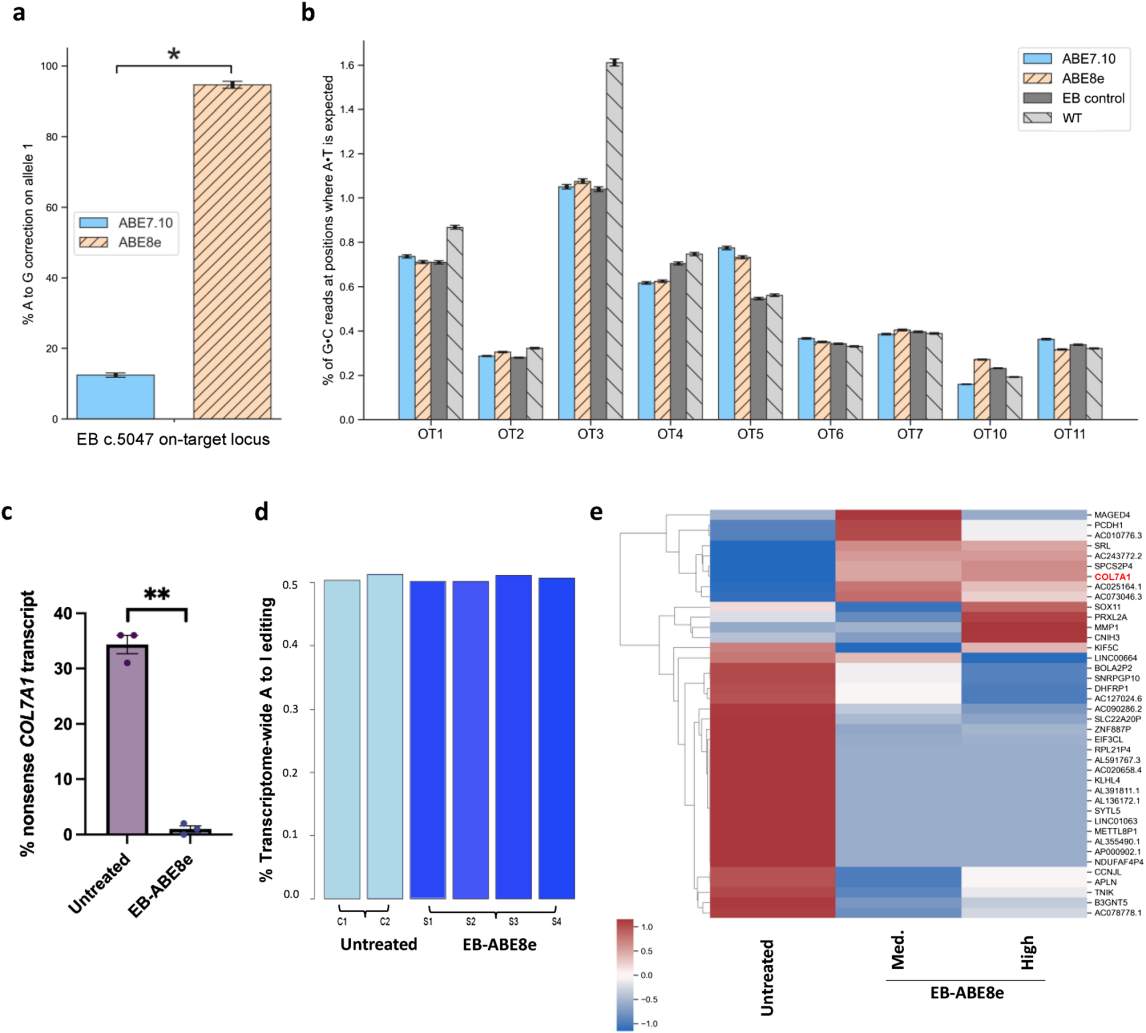
Assessment of effects of mutation correction on the transcriptome and genomic landscape. (**a**) NGS analysis of the on-target site found ABE7.10 corrected 12.4% of mutated alleles whereas ABE8e treatment led to 94.6% correction. Statistical difference and error bars were calculated using the Wald method. (**b**) 9 of the 10 predicted most likely sites for off-target editing across the genome were interrogated using NGS, illustrating negligible off-target editing activity following ABE7.10 and ABE8e treatment at all 9 sites. Error bars were calculated using the Wald method. (**c**) 0.5% of EB *COL7A1* RNA transcripts contained the nonsense mutation at c.5047 after treatment with ABE8e, compared to 36% without treatment. 3 technical replicates performed. Significance calculated using paired *t*-test (*p* = 0.0013) (**d**) ABE8e treatment led to no increase in A-to-I changes across the transcriptome (**e**) A heatmap generated from the log2 FPKM of the top 40 differentially expressed genes using the clustermap function of Python’s seaborn library, (version 0.11 https://seaborn.pydata.org/generated/seaborn.clustermap.html) is shown. Untreated EB fibroblasts were compared with fibroblasts treated with 5 µg sgRNA + 2 µg ABE8e (EB-ABE8e), as before, and to fibroblasts treated with 1 µg sgRNA + 2 µg ABE8e. The condition with 1 µg sgRNA + 2 µg ABE8e was termed EB-ABE8e Medium dose and the condition with 5 µg sgRNA + 2 µg ABE8e was termed EB-ABE8e High dose. *COL7A1* expression*,* highlighted in red, was increased after both treatments, with a greater increase following administration of EB-ABE8e High dose.

NGS was used to confirm on-target correction efficiency and found 94.6% on target editing in EB-ABE8e (Fig. 3a). The genome of the patient-derived fibroblasts was evaluated following editing by ABE8e or ABE7.10 to assess its propensity to convert A-T base pairs to G-C base pairs at off-target sites. Potential off-target loci were identified and ranked by CRISPOR^21^ software (http://crispor.tefor.net/) according to the validated Cutting Frequency Determination (CFD) score^22^ and the 10 most likely off-target genomic loci to be edited were selected (Fig. S2). Interrogation using NGS of these genomic loci in EB fibroblasts following 48 h exposure to ABE8e mRNA and sgRNA found no relevant off-target DNA editing (1% or less of G·C reads at positions where A·T is expected were detectable) at the 9 tested candidate off-target loci (6 genes and 3 intergenic regions) (Fig. 3b; Fig. S3). Testing of the remaining one predicted off-target site was not possible due to primer design. There was no difference in the degree of base pair conversions at off-target loci between ABE8e-treated, ABE7.10-treated and the untreated patient and WT conditions (Fig. 3b). Normal *COL7A1* transcripts were efficiently restored by ABE8e. We demonstrated 0.5% of RDEB *COL7A1* RNA transcript contained the mutated uracil at c.5047 after treatment with ABE8e, compared to 36% without treatment (Fig. 3c). The frequency of the mutated nucleotide is likely under 50% in untreated cells due to nonsense-mediated decay (NMD) of the mRNA. To uncover any unwanted effects at the RNA level after ABE8e correction, we performed transcriptome-wide RNA sequencing (RNA-seq) on ABE8e-treated and untreated patient fibroblasts. We assessed the frequency of adenine-to-inosine RNA deamination, a naturally occurring process from endogenous cellular deaminases^23^. ABE8e treatment led to no significant increase in A-to-I changes across the transcriptome relative to untreated fibroblasts (Fig. 3d). The transcriptome of untreated EB fibroblasts, which were not electroporated, were compared with fibroblasts treated with 5 µg sgRNA + 2 µg ABE8e (EB-ABE8e), as before, and also with fibroblasts treated with 1 µg sgRNA + 2 µg ABE8e. (Fig. 3e) The condition with 1 µg sgRNA + 2 µg ABE8e was termed EB-ABE8e medium dose and the condition with 5 µg sgRNA + 2 µg ABE8e was termed EB-ABE8e high dose to differentiate. 443 genes were significantly differentially expressed after treatment and heatmaps were generated from the log2 FPKM of the top 40 differentially expressed genes, ranked by the ToppGene tool^24^ (https://toppgene.cchmc.org/prioritization.jsp), and also all 443 genes (Fig. 3e; Fig. S4). *COL7A1* expression was increased after both treatments, with a greater increase following administration of EB-ABE8e high dose. Further analysis showed that ABE8e treatment of fibroblasts from the RDEB patient did not generate significant changes in the transcriptomic landscape relating to important cancer and metabolic pathways. (Fig. 3e; Fig. S4). Collectively, our findings indicate that ABE8e treatment of RDEB fibroblasts did not result in detectable off-target DNA or RNA editing using selected methods, despite high levels of on-target DNA editing.

### Editing efficiency is base editor and guide RNA dose dependent and correlates with C7 protein expression

**Figure 4:**
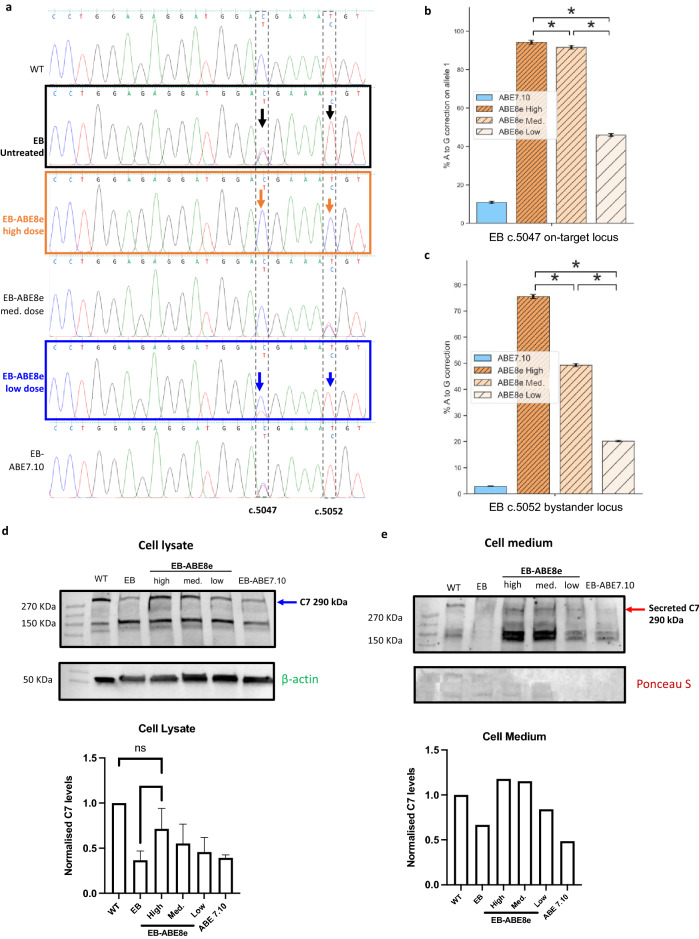
Altered doses of ABE8e and sgRNA treatment illustrate a dose-dependent relationship for bystander editing and protein restoration. (**a**) ABE8e and sgRNA were administered at high, medium and low doses and Sanger sequencing analysis performed post-editing to assess outcomes at mutation locus (c.5047) and the nucleotide in position 3 affected by bystander editing (c.5052). High dose = 5 µg sgRNA + 2 µg ABE8e, Med. dose = 1 µg sgRNA + 2 µg ABE8e, Low dose = 0.5 µg sgRNA + 1 µg ABE8e, ABE7.10 = 1 µg sgRNA + 2 µg ABE7.10. (**b**) NGS analysis of treatment outcomes at the c.5047 mutation locus found decreasing the High dose to Medium led to only a slight decrease in editing correction from 94.2 to 91.6%. The low dose engendered a correction of 45.9% of mutated alleles which was higher than ABE7.10 with an efficiency of 10.8%. Statistical difference and error bars were calculated using the Wald method. (**c**) NGS analysis of bystander editing at the c.5052 locus found that decreasing the ABE8e dose from High to Medium led to a large decrease in bystander editing, from 75.5 to 49.2%. Decreasing the ABE8e dose further resulted in 20.1% conversion of the bystander base, with ABE7.10 treatment causing 12.2% bystander editing. Statistical difference and error bars were calculated using the Wald method. (**d**) Western blots of protein collected from lysate of cells untreated and treated with different doses of ABE8e detected differential concentrations of a 290 kDa band, corresponding to full-length C7, using a polyclonal C7 antibody. Densitometric analysis of Western blots normalised to WT expression found EB cells treated by the highest dose of ABE8e expressed significantly more C7 in the cell lysate compared to untreated EB fibroblasts. There was no significance difference in C7 expression between the WT and the ABE8e High dose conditions nor between any of the untreated EB fibroblasts and the ABE8e Medium dose, Low dose and ABE7.10 conditions. N = 4 for WT, EB and ABE8e High dose conditions. N = 3 for ABE8e Medium dose and N = 2 for ABE8e low dose and ABE7.10 conditions. Representative blot shown. Significance found using the *T*-test. Cropped blots shown, full blots displayed in Supplementary Fig. S5a,b,e Western blots of cell medium collected from untreated and treated cells and probed by the polyclonal C7 antibody detected a 290 kDa band corresponding to secreted full-length C7. Densitometric analysis normalised to WT expression levels found EB cells treated by the highest and medium doses of ABE8e secreted similar C7 levels to Wild Type fibroblasts (WT) and more than untreated EB cells. EB fibroblasts treated with the low dose of ABE8e engendered a marginal increase in C7 whereas expression after ABE7.10 treatment was largely unchanged. n = 1. Full blots of C7 and Ponceau staining displayed in Supplementary Figs. S5c and S5d.

Bystander mutations can occur within the editing window when using base editors^25^. Despite the high correction rate of c.5047 C > T mutation in *COL7A1*, editing with ABE8e caused a silent bystander mutation together with correction of the pathogenic nonsense mutation. We hypothesized that the editing efficiency and occurrence of the bystander mutation are proportional to the doses of ABE8e and sgRNA used in this study and that altering these doses could help diminish the bystander editing. We tested different doses to determine whether optimal conditions could be found in which the editing efficiency of the nonsense mutation remained at a clinically relevant level yet the bystander mutation was reduced. ABE8e and sgRNA were administered at high (5 µg sgRNA + 2 µg ABE8e), medium (1 µg sgRNA + 2 µg ABE8e) and low (0.5 µg sgRNA + 1 µg ABE8e) doses and Sanger sequencing analysis assessed outcomes post-editing at the mutation locus (c.5047) and the nucleotide in position 3 affected by bystander editing (c.5052). We confirmed 100% mutation correction with the high dose, along with a 98% bystander mutation at c.5052. Switching to the medium dose maintained 100% correction yet reduced the bystander mutation to 56%. Using the low dose, the editing efficiency was 64% while the bystander mutation was reduced to 2%. Of note, ABE7.10 had a lower editing efficiency of 8% but created no bystander mutation (Fig. 4a). As a more sensitive measure, NGS analysis of treatment outcomes was also employed. This analysis showed that decreasing the high dose to the medium dose led to only a slight decrease in editing correction at the treatment locus from 94.2 to 91.6% but resulted in a large decrease in bystander editing, from 75.5 to 49.2%. The low dose engendered a correction of 45.9% of mutated alleles and 20.1% conversion of the bystander base. ABE7.10 had an efficiency of 10.8%, with bystander editing below 5% (Fig. 4b,c). Western blots of protein collected from the cell medium and lysate of cells treated and untreated with ABE8e detected differential concentrations of a ~ 290 kDa band, corresponding to full-length C7 (Fig. 4d,e). Densitometric analysis of western blots showed RDEB cells treated with the highest dose of ABE8e expressed significantly greater quantities in the cell lysate compared to untreated EB fibroblasts, an increase of approximately twofold (Fig. 4d). There was no significant difference in the C7 levels between WT and EB cells corrected by the highest dose of ABE8e. Treatment at the highest and medium doses also led to elevation of C7 levels in the cell medium to similar levels in WT (Fig. 4e). In cells treated with the low dose and ABE7.10, expression levels of C7 in the cell medium and lysate were largely unchanged (Fig. 4d,e). This can be attributed to the modest gene correction efficiency by the low dose of ABE8e and ABE7.10.

### Collagen VII deposition is present in 3D skin equivalents generated using base edited with ABE8e RDEB patients derived fibroblasts

**Figure 5:**
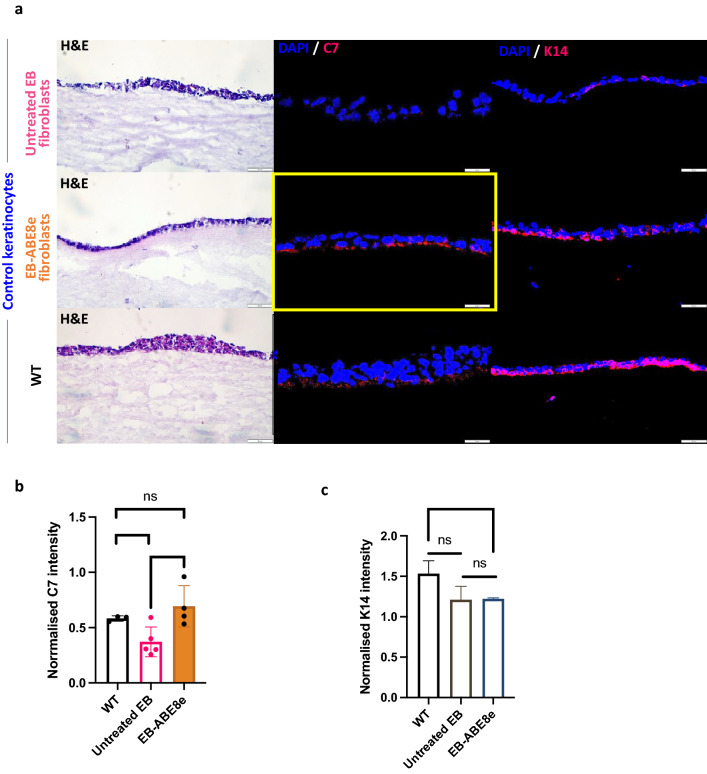
Histology and expression of collagen type VII (C7) and keratin 14 (K14) in skin constructs containing either untreated EB, ABE8e treated or Wild Type fibroblasts (WT) in combination with control keratinocytes. (**a**) Staining for C7 using LH7.2 monoclonal antibody reveals that gene editing of EB fibroblasts with the high dose of ABE8e base editor restored C7 expression at the dermal–epidermal junction in skin equivalent cultures created with healthy control keratinocytes. Images of skin constructs cryosections have been taken under bright light (H&E staining) or fluorescent microscope (C7 and K14 expression in immunofluorescent staining with DAPI showing nuclei); scale bars measuring 50 µm. (**b**) Quantification of C7 intensity normalised by cell number found a significant increase in C7 intensity between untreated EB and EB-ABE8e and no significant difference in C7 intensity between WT and treated cells. *t*-test performed. (**c**) Quantification of K14 intensity normalised by cell number found no significant difference between WT and untreated EB or untreated EB and EB-ABE8e however there was a significance difference between WT and EB-ABE8e K14 intensity. *t*-test performed.

Encouraged by these findings, we tested if RDEB fibroblasts, following base editing with ABE8e, can re-express C7 in 3D human skin equivalents (HSEs). The 3D HSE model comprises combined tissue culture of keratinocytes and fibroblasts, generated as previously reported^26^. After 20 days of maturation, the HSEs were harvested, and we used histology to examine the expression of C7 and the basal keratinocyte marker keratin 14 (K14) (Fig. 5a). Relative fluorescence intensities of the two proteins were quantified and normalised to the cell number. Quantification analysis revealed that HSEs constructed with untreated EB fibroblasts produced the lowest C7 intensity whereas HSEs composed of ABE8e edited fibroblasts significantly increased C7 expression akin to wild type HSEs (Fig. 5b). There was no significant difference in K14 intensity between HSEs composed of untreated and EB-ABE8e treated fibroblasts, therefore controlling for keratinocyte C7 production (Fig. 5c). There was a significantly greater intensity of K14 expression in WT compared to the EB-ABE8e HSE (Fig. 5c).

## Discussion

The treatment of rare inherited diseases such as RDEB could be revolutionized by the ability to reverse the root genetic cause, potentially providing one-time cures. Our gene editing data indicate that base editing of *COL7A1* using ABE8e is extremely efficient and can improve skin function in this blistering skin disease. Base editors such as ABE are recently-developed gene editing tools capable of precisely installing point mutations without causing double stranded cleavage or relying on HDR and a donor DNA template^13,27^. Here, we achieved 94.6% correction of alleles harboring one of the most recurrent *COL7A1* mutations that causes RDEB using ABE8e^18^. ABE8e performed significantly better than ABE7.10 which only corrected 12.4% of mutated alleles. This better gene editing performance was expected as the deaminase in ABE8e was enhanced through phage-assisted non-continuous and continuous evolution^18^ to introduce eight additional mutations in the TadA* domain. In our study, ABE8e engendered markedly higher levels of correction in primary fibroblasts than has been achieved in previous work using CRISPR-Cas9 or older versions of ABE^16^. ABE8e therefore appears to be a powerful tool for correcting pathogenic *COL7A1* point mutations.

By demonstrating successful treatment of almost every cell in a bulk population of patient-derived fibroblasts, this work has advantages for clinical translation. First, antibiotic or fluorescence-based selection markers were not required to enrich for corrected primary cells, as in previous studies^28^. Secondly, the high efficiency also obviated the need to generate patient-derived induced Pluripotent Stem Cells (iPSCs) to isolate purely corrected clones, reducing the time and costs required and avoiding concerns related to tumorigenicity and immunogenicity when deploying iPSCs clinically^26^.

After ascertaining correction of the mutation in the genome, targeted RNA sequencing confirmed that mutated *COL7A1* transcripts had been replaced by wild-type transcripts following treatment with ABE8e. This correction was also evident in the proteins expressed by the cells measured by western blot and histology of 3D human skin equivalents. These observations give promise that the levels of genomic DNA correction achieved using ABE8e in our study will be sufficient to generate AF formation in vivo as previous studies suggest as little as 26% of normal C7 levels are required for such architectural recovery^28^. The structural restoration of AF, however, will be determined in our future work alongside the correction of patient-derived cells with other *COL7A1* mutations to promote its wider applicability. A key limitation of base editing remains its restriction to correcting transition point mutations and its strict PAM site requirements^10^. Insertion and deletion mutations in *COL7A1*, however, could be corrected by employing complementary gene editing tools such as prime editors which also do not cause DSBs^29^. Furthermore, a suite of BEs which now recognise non-NGG PAM sites can be utilised to target a wider range of *COL7A1* mutations in future work.

Safety, alongside efficiency, is a critical requirement for targeted therapies for RDEB. A major limitation for gene editing strategies thus far has been concerns relating to off-target editing which could cause genotoxicity or oncogenic transformation. Base editors have been shown to exhibit off-target activity both in the genome and the transcriptome, therefore we characterized these changes in treated and untreated cells. Analysis of 9 off-target genomic loci predicted to most likely be edited found no significant changes in the ABE8e-treated cells. The nature of ABEs to target the transcriptome has been recognised previously^30^ and our study uncovered 443 differentially expressed genes following ABE8e treatment. Of note, there were no significant discrepancies relating to important cancer and metabolic pathways when comparing base edited to non-edited RDEB fibroblasts. Furthermore, transcriptome wide analysis determined there was no increase in A-I changes following editing meaning it is possible differential expression changes were induced by cell culture, which has been found to significantly alter gene expression signatures^31^. Mutations introduced into the TadA domain of ABE8e have been shown to further decrease its RNA editing profile which can be incorporated into future translational work^32^. Nevertheless, our results, together with previous studies, demonstrate encouraging safety data for the use of ABEs in the clinic^12,33–35^.

Other adenine nucleobases in the editing window of ABEs are liable to be converted to guanine in addition to the target base, an effect known as ‘bystander editing’^25,36^. We discovered bystander editing had occurred after treatment based on genomic DNA sequencing. Although this bystander edit does not cause a change in the coding sequence of *COL7A1*, we sought to reduce the bystander effect to maximize the clinical applicability of our approach. By adjusting the doses of the ABE8e and sgRNA, the bystander editing was reduced to just 20.1% of alleles with 49.2% correction of the mutation. Other work has pioneered methods of reducing bystander editing aside from reducing concentrations of ABE8e and sgRNA. Very recently, combinatorial engineering has generated ‘NG-ABE9e’, an adenine base editor which recognises an ‘NGN’ PAM site and exhibits a seven to fourfold reduction in bystander editing from ABE8e, whilst maintaining comparable efficiency^37^. A version which includes the ‘D108Q’ mutation in the ABE8e coding sequence also reduces any bystander deamination of cytosine^38^. Novel ABE versions can be included in our future studies to preclude bystander editing without compromising efficiency. Furthermore, to obviate cumbersome experimental screening of different base editors, machine learning algorithms can predict base editing outcomes across the protospacer sequence and therefore the degree of likely bystander editing. 3 robust algorithms are the ‘BEHive10’^39^, ‘DeepBaseEditor13’^40^ and the 'BE-DICT’^41^ prediction tools which will prove useful during ongoing work.

Base editing with a high dose of adenine base editor did not generate numerous off-targets single-nucleotide polymorphisms (SNPs), consistent with other studies^17,35,38,42–44^. Naturally, some off-target edits may not have been detected using our strategy and future work will use whole-genome sequencing and specific oncogene panel screening prior to clinical translation^45,46^.

We selected mRNA instead of plasmid DNA as the form to deliver base editors because its rapid but transient expression has been reported to decrease bystander and off-target editing, circumvent the risk of plasmid DNA random integration in the genome, and result in better overall editing efficiency^47–50^. Clinical translation of gene editing therapies using an in vivo mRNA (mRNA) system for DEB is also ideal. mRNA has gained traction as a class of therapeutic agent to target various diseases, with one option for therapeutic delivery of mRNAs being the use of lipid nanoparticles (LNPs). Notably, lipid nanoparticle-mRNA vaccines are in widespread clinical use for COVID-19, marking a milestone for mRNA therapeutics^51–55^. Therefore, our future work testing the delivery of ABE8e mRNA in combination with LNP technology for RDEB has great potential. mRNA-based gene editing therapeutics can be produced in an inexpensive and scalable manner through synthetic manufacturing making it an attractive platform to develop novel, cost-effective treatments for RDEB and additional inherited disorders affecting skin and other organs.

## Materials and methods

### Ethics statement

All methods were carried out in accordance with the Declaration of Helsinki. The gene editing studies were approved by HRA and Health and Care Research Wales (HCRW) Approval (IRAS project ID: 288555).

### Primary fibroblast culture

Primary patient fibroblasts (EB fibroblasts) isolated from punch biopsies, were obtained from the cell bank at St. John’s Institute of Dermatology. EB fibroblasts were cultured in Dulbecco’s Modified Eagle Medium (DMEM), (Gibco), supplemented with 10% Fetal Bovine Serum (FBS), (Gibco), and 1% penicillin/streptomycin, (Invitrogen). The fibroblasts were grown at 37 °C and 5% CO_2_ and routinely passaged using TrypLE™ Express (Gibco) as a gentle trypsin replacement enzyme to detach and dissociate the cells.

### sgRNA design

The sgRNA targeting the c.5047 C > T mutation of the *COL7A1* gene were designed using the CRISPR RGEN guide-RNA designer tool for base editing^19^ (http://www.rgenome.net/be-designer/). The guides were manufactured as synthetic sgRNA chemically modified molecules by Synthego and Invitrogen.

### Base editors

ABE7.10 mRNA was obtained from Dr. Anastasia Petrova at the UCL GOS Institute of Child Health. ABE8e was obtained from Dr. David Liu’s lab at the Broad Institute of Harvard and MIT and used in the preliminary base editing experiment to compare the editing efficiency between both ABEs. ABE8e mRNAs included full substitution of uracil for N1-methylpseudouridine, co-transcriptional 5’ capping with the CleanCap AG analog resulting in a 5’ Cap1 structure, and included a 120 nucleotide polyA tail. For the follow up gene editing experiments to correct the mutations, ABE8e mRNA synthesised by TriLink was used.

### Electroporation

The Neon™ Transfection System MPK5000 (Invitrogen) was used to electroporate the fibroblasts with the base editor and sgRNA. The base editing reagents (sgRNA and ABE) for each condition were prepared in a separate well of a 96-well plate and made up to a total volume of 10 μl with TE buffer. 1 × 10^6^ Fibroblasts were pelleted (1200 rpm, 5 min), and resuspended in 100 μl Resuspension Buffer R (Neon kit) per condition just before electroporation. 100 μl of the cell suspension was mixed with the base editing reagents, taken up in a 100 μl Neon™ tip ensuring there is no bubble at the top, placed in the pipette holder containing 3 ml of Buffer E2 (Neon kit) and electroporated at Voltage = 1500 V; Width = 20 ms; # pulses = 1 pulse.

### DNA/RNA isolation

Genomic DNA was isolated using the QIAamp DNA Mini Kit (Qiagen) and RNA was isolated using the RNeasy Kit (Qiagen) according to manufacturer’s protocols. The DNA and RNA were eluted with 40 μl of elution buffer AE and RNase-free water respectively at the end of the extraction process. The concentration and purity of the nucleic acid was measured on a NanoDrop 1000 Spectrophotometer (Thermo Scientific) at the A260/A280 absorbance ratio appropriate for nucleic acids.

### PCR amplification

The relevant DNA fragment containing the mutations of interest was amplified using polymerase chain reaction (PCR). 50–100 ng of DNA were used as starting material for all PCR amplifications. A PCR master mix was made using AmpliTaq GoldTM 360 DNA polymerase (Applied Biosystems), GC buffer (Applied Biosystems), primers at 10 mM concentration (Invitrogen) and nuclease-free water (Synthego). The following primers used for NGS analysis are listed in Table 1.

**Table 1:** Primers used for NGS analysis.

Target	Primer	NGS primer sequence (5’–3’)
On-target	C7_53_NGS_fwd	**CGTCGGCAGCGTCAGATGTGTATAAGAGACAG**CTGCTGCTCAGACCCTTCTC
	C7_54_NGS_rev	**GTCTCGTGGGCTCGGAGATGTGTATAAGAGACAG**GCCTTCAGATGCGTGTGTGC
Off-target	1OT_NGS_fwd	**TCGTCGGCAGCGTCAGATGTGTATAAGAGACAG**CGAGATGATAGGGAGGCAAAG
	1OT_NGS_rev	**GTCTCGTGGGCTCGGAGATGTGTATAAGAGACAG**TGTTTGTGGTGGACTCGGC
	2OT_NGS_fwd	**TCGTCGGCAGCGTCAGATGTGTATAAGAGACAG**CAAACTCCCCTGCTGACCTC
	2OT_NGS_rev	**GTCTCGTGGGCTCGGAGATGTGTATAAGAGACAG**CCCTCCCTGCAGATTCCAAG
	3OT_NGS_fwd	**TCGTCGGCAGCGTCAGATGTGTATAAGAGACAG**GCGTCTGTAGAGCCGATACC
	3OT_NGS_rev	**GTCTCGTGGGCTCGGAGATGTGTATAAGAGACAG**CCCACTTTTCCCAGGCATT
	4OT_NGS_fwd	**TCGTCGGCAGCGTCAGATGTGTATAAGAGACAG**GGGGGATGAGGGCAGAATTT
	4OT_NGS_rev	**GTCTCGTGGGCTCGGAGATGTGTATAAGAGACAG**TTTGGGGGTCCAGGAGGAAT
	5OT_NGS_fwd	**TCGTCGGCAGCGTCAGATGTGTATAAGAGACAG**TGGTTCCCGGTTGTCTATGG
	5OT_NGS_rev	**GTCTCGTGGGCTCGGAGATGTGTATAAGAGACAG**GAGTCTTGTGGAAGGTCTTTA
	6OT_NGS_fwd	**TCGTCGGCAGCGTCAGATGTGTATAAGAGACAG**CTACTCAGGAGGCTGAAGC
	6OT_NGS_rev	**GTCTCGTGGGCTCGGAGATGTGTATAAGAGACAG**AGGGCAGGTGAAAGGAAGGC
	7OT_NGS_fwd	**TCGTCGGCAGCGTCAGATGTGTATAAGAGACAG**CTCATAACTCCCACAACAGG
	7OT_NGS_rev	**GTCTCGTGGGCTCGGAGATGTGTATAAGAGACAG**AGCCAGCAACATTGACCTCT
	10OT_NGS_fwd	**TCGTCGGCAGCGTCAGATGTGTATAAGAGACAG**CCCAGAGGATCACCTTTCCC
	10OT_NGS_rev	**GTCTCGTGGGCTCGGAGATGTGTATAAGAGACAG**CAACCCTGAGAGACAGGTGC
	11OT_NGS_fwd	**TCGTCGGCAGCGTCAGATGTGTATAAGAGACAG**TCACACCCAGAAATGGAGCC
	11OT_NGS_rev	**GTCTCGTGGGCTCGGAGATGTGTATAAGAGACAG**AGGCAAGGGAAACTTAGGCAA

### Sanger sequencing

The PCR products were purified from unincorporated primers and dNTPs using illustraTM ExoProStarTM (Cytiva). The reaction was heated at 37 °C for 30 min, 80 °C for 15 min, and held at 10 °C using the Veriti Thermo cycler. Samples were sequenced externally (SourceBioScience) and the result chromatograms were analyzed using SnapGene viewer. Editing efficiency was analysed using EditR software^20^ (http://baseeditr.com/).

### NGS genomic DNA on-target site analysis

The same DNA samples used in the Sanger sequencing method were subjected to high-throughput sequencing (Next-Generation Sequencing, NGS) with sequence-specific primers with NGS overhang, overhang shown in bold in Table 1. All sequencing runs were performed on a MiSeq instrument (Illumina Co), using MiSeq Reagent Kit v2 nano (500 cycles) following the manufacturer’s protocol. The sequences were obtained in fastq format with demultiplexing performed automatically. Sequence annotation to the human genome (hg38) was done in Bowtie2^56^, obtaining the .sam files. Converting .sam files to .bam and sorted bam files was undertaken using SAMtools^57^ (http://www.htslib.org/). The counts of the individual base reads against genome positions for further calculations was executed using Pysamstats (https://github.com/alimanfoo/pysamstats). A 23-nucleotide window corresponding to the protospacer sequence and the PAM site was selected for each sample. A detailed analysis of reads was then performed at position 8 in the protospacer, which corresponded to the position of the mutation on a positive strand, and at position 3 on the positive strand, which corresponded to the bystander mutation detected earlier using Sanger sequencing. Custom Python scripts (see Github repository) were used to calculate the editing efficiency and off-target sequence analysis. Graphs were created with the use of the Matplotlib library for creating graphs for the Python programming language and its numerical extension NumPy, and the data visualization library Seaborn.

The efficiency of the editing “on-target c.5047”, chr3: 48,580,586 was calculated as follows:

$$c.5047A>G\, editing\left[\%\right]=\frac{G\, reads\, at\, pos. \,48580586}{all\, reads\, at\, pos.\, 48580586}\times 100\%$$

$$efficiency\left[\%\right]=\frac{\left(c.5047A>G\, editing\left[\%\right]\right) -\left(EB\, control\, ref.\, G\, reads\, at\, pos.\, 48580586\left[\%\right]\right)}{100\% - \left(EB\, control\, ref.\, G\, reads\, at\, pos.\, 48580586\left[\%\right]\right)}\times 100\%$$

The frequency of the “silent bystander c.5052”, chr3: 48,580,581 was calculated as follows:

$$c.5052A>G\, editing\left[\%\right]=\frac{G\, reads\, at\, pos.\, 48580581}{all\, reads \,at\, pos. \,48580581}\times 100\%$$

Normalization required for dose-dependent experiment results NGS sequencing (due to artifact G reads at pos. 48,580,581 for EB control:

$$bys.\,freq \left[\%\right]=\frac{\left(c.\,5052A>G\, editing\left[\%\right]\right) -\left(EB \,control\,ref.\, G \,reads\, at\, pos. \,48580581\left[\%\right]\right)}{100\% - \left(EB \,control \,ref. \,G\, reads\, at \,pos.\, 48580581\left[\%\right]\right)}\times 100\%$$

### NGS genomic DNA off-target site analysis

Amplicons containing the 23-nucleotide sequence of a potential off-target were amplified as above. The 10 most likely genome-wide off-target sites to be edited were computed using CRISPOR software^21^. Out of the initial 10 off-targets suggested, OT9, on the Y chromosome, could not be interrogated as patient EB is female, therefore the list of 10 was extended by an additional 1). OT8 could not produce an amplicon of the correct size due to the reagents selected for NGS (Next-Generation Sequencing), therefore it was also omitted from analysis. For the 9 remaining off-target amplicons, positions 3 to 9 of the protospacer (shown underlined in supplementary Fig. 2 (Fig. S2)), which corresponds to the extended putative editing window, were analyzed as described above for the on-target site.

$$cumulative\; G\cdot C>A\cdot T\left[\%\right]=\frac{sum\; of\; changes\; G>A\; \& \;C>\;T\; at\; pos. \;from \;3\; to \;9 \;of \;protosp.}{sum\; of\; ref.\; A\; \& \;T \;at\, pos.\, from \;3\; to\; 9 \;of \;protosp.}\times 100\%$$

To compute 95% confidence intervals for each correction, bystander and off-target activity, which are shown in figures, we used Wald method, a commonly used approximation for binomial confidence intervals. We used this method to estimate errors because the experiments and sequencing of DNA was performed in one repetition. We are aware that this error estimation method does not capture their other sources.

### Transcriptomic analysis

Total mRNA was isolated using standard methods. RNA-Sequencing libraries were prepared by KAPA RNA Hyper + RiboErase HMR Kit (Roche Corp.) according to the manufacturer’s standard protocol. The quality of the library was checked by TapeStation electrophoresis (Agilent Technologies). Libraries were then sequenced on the NextSeq 500/550 instrument (Illumina Co) with High Output Kit v2.5 following the manufacturer’s protocol. Image processing, base calling and demultiplexing were made by NextSeq Control Sofware (Illumina Co). FASTQ-formatted sequences were analysed for quality control by FASTQC open-source software http://www.bioinformatics.babraham.ac.uk/projects/fastqc/. Reads with quality scores below 24 were excluded. The HISAT2 software^58^ as used to align reads to the human reference genome (Hg38). SAMtools software were used to convert .sam to .bam files and the featureCounts algorithm was used to count the mapped readings (from .bam file). This general purpose read summary function assigned genomic traits (or meta-traits) to the mapped reads which were generated from RNA sequencing. The featureCounts algorithm performed transcript assembly, quantification of expression as fragments per kilobase of transcript per million mapped reads (FPKM) values, and normalization. Normalized FPKM values were used for the differential expression analysis, which was performed using an ‘edger’ R script. The clustermap function from Python’s seaborn^59^ library (https://seaborn.pydata.org/generated/seaborn.clustermap.html, version 0.11) was used to create the heatmap. The clustermap uses hierarchical clustering^60^ implemented in the scipy library^61^. Candidate genes which were differentially expressed between the ABE8e-treated cells and the untreated cells were then selected and the ToppGene tool^24^ was used to prioritize and rank those genes based on functional similarity to the training gene list (which was obtained from GeneCard tool). The output of the Database for Annotation, Visualization, and Integrated Discovery (DAVID) bioinformatics tool revealed that these genes are members of 40 pathways. A *p*-value of < 0.05 was used to indicate statistical significance.

To calculate the mean % A to I change over the entire transcriptome, we used REDItools v1.3^62^ to quantify the % edit in each sample. This tool removes all nucleotides from the analysis, except adenosine, and then removes all adenosines with read ranges less than 10 to avoid errors due to low trying. The items with mapping or reading of the quality score below 25 were then removed. The number of adenosines converted to inosine in each sample was then calculated and divided by the total number of adenosines in the data set after filtering to obtain a percentage of adenosine edited to inosine in the transcriptome.

### Cell culture and protein extraction

Fibroblasts were seeded at 1 × 10^5^ density in a 6 well plate initially in DMEM medium with 10% FBS. After 24 h, the cells were gently washed with PBS, and grown in Opti-MEM (+ 1% P/S), (Gibco) supplemented with 50 µg/ml ascorbic acid (5% v/v) for an additional 48 h. The supernatant was collected, diluted 1:4 with ice-cold acetone, and stored at − 20 °C overnight. Supernatant was cleared of any cell debris by centrifuging at 500 rotational centrifugal force (RCF) for 5 min and transferred to a new falcon. Supernatant proteins were collected by centrifugation at 13,000 rpm for 10 min. Following centrifugation, the supernatant was discarded, and the pellet was dried on ice for 10–15 min, without letting it to fully dry. The pellet was then dissolved in 100 μl sample buffer by vortexing and boiled at 95 °C for 10 min. To extract protein from the cells, the well was washed with 1 × PBS (Gibco) before adding 100 μl lysis buffer (1% β-mercaptoethanol, 1% Proteinase Inhibitor Cocktail (Calbiochem), 25% 4X NuPAGE LDS sample buffer (Invitrogen) and 75% H2O). The cells were scraped and left in a 4 °C cold room for 30 min. The lysis extract was collected in a 1.5 ml tube, centrifuged at 13,000 rpm for 2 min to remove cell debris, and the supernatant was transferred to a fresh 1.5 ml tube and boiled at 95 °C for 10 min. All protein samples were stored at − 20 °C.

### Protein quantification

The Pierce™ BCA (bicinchoninic assay) Protein Assay Kit (Thermo Scientific) was used according to the manufacturer’s instructions to quantify the total amount of protein in each sample and normalise the amount of sample loaded into the gel. The only deviation from the original protocol was halving the reaction volume to prevent spillage due to overflow in the wells. The absorbance was measured using the UV/Vis setting on the FLUOstar Omega Microplate Reader (BMG LABTECH) and used to quantify the amount of protein present in each sample.

### Western blot

Normalized samples were loaded into a 4–20% Sodium Dodecyl Sulphate Polyacrylamide Gel (Bio-Rad), along with Spectra™ Multicolour High Range Protein Ladder (Thermo Scientific) and run at 80 V for 70–80 min. Following Sodium Dodecyl Sulphate Polyacrylamide Gel Electrophoresis (SDS-PAGE), the proteins were transferred onto a PVDF membrane (Bio-Rad) using the Turbo Transfer (Bio-Rad) at 25 V for 10 min. The total protein was visualized using reversible Ponceau Solution staining (Sigma-Aldrich). The membrane was then incubated with a rabbit anti-collagen VII polyclonal primary antibody (Bio-Rad, VPA00854) diluted 1:1000 in 5% Bovine Serum Albumin (BSA) in TBS-1% Tween (TBS-T, Bio-Rad) overnight at 4 °C. Secondary anti-rabbit antibody, conjugated with polyclonal horseradish-peroxidase (HRP) (Dako) diluted 1:2000 in 5% BSA in TBS-T, was incubated for 30 min at room temperature. β-actin mouse primary antibody (SantaCruz Biotechnologies) diluted 1:1000 in 5% BSA in TBS-T and goat anti-mouse conjugated with polyclonal HRP (Dako) was used as a housekeeping gene for protein normalization in the cell lysate samples. SuperSignal™ West Femto Maximum Sensitivity Substrate (Thermo Scientific) was used as a substrate before imaging using iBright equipment (Applied Biosystems). Quantification of the bands was done using ImageJ software^63^ (version 1.53t, https://imagej.nih.gov/ij/) and normalized against β-actin or Ponceau S.

### Immunofluorescence

Fibroblasts were plated on round 13 mm coverslips at a density of 10,000 cells/cm^2^ in a 24-well tissue culture plate and grown for 48 h. Once ~ 60% confluent, the cells were washed with 1 × PBS and fixed with 4% paraformaldehyde for 20 min at room temperature (RT). Subsequently, the cells were permeabilized for 20 min at RT with 0.2% TritonX-100 (Merck). The coverslips were incubated with a primary antibody diluted in 5% BSA in PBS overnight at 4 °C. The following day, the cells were washed three times with 1 × PBS, incubated with the respective donkey anti-rabbit (Alexa Fluor 488), goat anti-mouse (Alexa Fluor 488) or goat anti-mouse (Alexa Fluor 594) secondary antibody for 2 h at RT, and washed again three times with 1 × PBS. The coverslips were then mounted on glass slides using mounting medium that contains 4′,6-diamidino-2-phenylindole (DAPI) nuclear stain (Vectashield) and imaged using an BX53M industrial microscope (Olympus).

### 3D skin equivalents

3D human skin equivalents (HSEs) were generated similarly to methods described previously^64^.

## Supplementary Information

Supplementary Information.

## Data Availability

Gene expression datasets generated and analysed during the current study are available in the Gene Expression Omnibus (GEO) repository, GSE211876 https://www.ncbi.nlm.nih.gov/geo/query/acc.cgi?acc=GSE211876 All other datasets are available from the corresponding author on reasonable request.

## Code availability

The code used in this article was deposited in GITHUB repository (https://github.com/LabGiB/base_editor_offtarget).
