# Supplementary material for: ABE8e adenine base editor precisely and efficiently corrects a recurrent COL7A1 nonsense mutation
Source: Sci Rep. 2022 Nov 16;12:19643. doi: 10.1038/s41598-022-24184-8 (PMC9666996; doi:10.1038/s41598-022-24184-8)
Supplement: Supplementary file 1 — Supplementary Information. [file 41598_2022_24184_MOESM1_ESM.docx]

**Supplementary Figures: ABE8e adenine base editor precisely and efficiently corrects a recurrent *COL7A1* nonsense mutation**

Adam Sheriff^1^, Ina Guri^1^, Paulina Żebrowska^2^, Virginia Llopis-Hernandez^1^, Imogen R. Brooks^1^, Stavroula Tekkela^1^, Kavita Subramaniam^1^, Ruta Gebrezgabher^1^, Gaetano Naso^3^, Anastasia Petrova^3^, Katarzyna Balon^2^, Alexandros Onoufriadis^1^, Dorota Kujawa^2^, Martyna Kotulska^2^, Greg Newby^4,5,6^, Łukasz Łaczmański^2^, David R. Liu^4,5,6^, John A. McGrath^1^, Joanna Jacków^1*^

^1^ St John’s Institute of Dermatology, King’s College London, London, UK

^2^ Hirszfeld Institute of Immunology and Experimental Therapy, Polish Academy of Sciences, Poland, PL

^3^ Molecular and Cellular Immunology Unit, UCL GOS Institute of Child Health, London, UK

^4^ Merkin Institute of Transformative Technologies in Healthcare, Broad Institute of Harvard and

MIT, Cambridge, MA, USA

^5^Department of Chemistry and Chemical Biology, Harvard University, Cambridge, MA, USA

^6^Howard Hughes Medical Institute, Harvard University, Cambridge, MA, USA

*Correspondence should be addressed to:

Dr Joanna Jacków, Ph.D

Lecturer in Molecular Gene Expression and Gene Editing

St John’s Institute of Dermatology

Faculty of Life Sciences & Medicine

King's College London

9th Floor Tower Wing

Guy’s Hospital

Great Maze Pond Road, London SE1 9RT, UK

Telephone: +44(0) 20 7188 9613

Email: [joanna.jackow@kcl.ac.uk](mailto:joanna.jackow@kcl.ac.uk)

**Short title:** Base editing for recessive dystrophic epidermolysis bullosa

**Key words:** collagen VII, recessive dystrophic epidermolysis bullosa, base editing, off-target

**Supplementary Figure S1 (a)** RT-PCR Transcript analysis of COL7A1 Exons 53-54 and Exon/Intron 95. EB patient cells exhibit reduced transcripts of Exon 53-54 compared to WT due to nonsense mediated decay of the mutated transcript from Allele 1 which harboured the c.5047 C>T mutation. EB patient cells also have an extra smaller sized band of the Exon/Intron 95 transcript compared to WT which was produced by Allele 2 due to to the exon-skipping mutation c.7344+1 G>A. **(b)** Full Western blot of secreted type VII collagen (C7) levels in pre-treated EB fibroblasts compared to Wild-type (WT) fibroblasts. Full-length C7 detected in cell medium using a C7 antibody**. (c)** Full Western blot of intracellular type VII collagen (C7) levels in pre-treated EB fibroblasts compared to Wild-type (WT) fibroblasts. Full-length type VII collagen detected in cell lysates using a C7 antibody. **(d)** Full Western blot of protein in the cell medium stained by Ponceau S. **(e)** Full Western Blot of protein in the cell lysate stained by Ponceau S.

**Figure S2 11 putative off-target (OT) sites selected by CRISPOR tool sorted by declining probability of editing.** The underlined nucleobases are those in the editing window that were considered in the off-target calculations. The on-target Adenine nucleobase on the positive strand at position c.5047 (chr3: 48580586) is highlighted in green. Highlighted in yellow is the bystander Adenine nucleobase at position c.5052 (chr3: 48580581). For each OT, where they are in the protein coding region (exon or intron), the protein name and its main functions are described. Descriptions for the intergenic regions are omitted.

**Figure S3. (a) Logarithmic scale comparison of bystander and off-target editing activity in EB-ABE8e, EB-ABE7.10 and untreated EB and WT cells.** The percentage of C·G reads where A·T is expected was normalised by the baseline (54.03% of C at c.5047 in EB cells)**.** The bystander mutation at position c.5052 and the 9 of the 10 predicted most likely sites for off-target editing across the genome were interrogated using NGS, illustrating negligible off-target editing activity following ABE7.10 and ABE8e treatment at all 9 sites and a higher efficiency of mutation introduced by ABE8e compared to ABE7.10 at the bystander mutation. **(b)** Height of the bars (%) at the c.5052 position and the 9 OTs, collated in a table.

**Figure S4. Full heatmap of 443 differentially expressed genes and regions detected (p<0.05).** Following RNA-Seq, normalized fragments per kilobase of transcript per million mapped reads (FPKM) were used to shortlist genes which were significantly differentially expressed, using a p-value of < 0.05 which yielded 443 differentially expressed genes. This was compared between untreated EB cells, EB-ABE8e high dose and EB-ABE8e medium dose. A heatmap generated using the clustermap function (https://seaborn.pydata.org/generated/seaborn.clustermap.html) from Python’s seaborn library from the log2 FPKM of all 443 differentially expressed genes is shown.

**Supplementary Figure S5.** **(a)** Full Western Blot of WT, untreated and treated fibroblasts. Polyclonal antibody used to stain for C7 in the cell lysate. **(b)** Full Western Blot of WT, untreated and treated fibroblasts. Monoclonal antibody used to stain for C7 in the cell lysate to normalize differential values of C7 intensity. **(c)** Full Western Blot of WT, untreated and treated fibroblasts. Polyclonal antibody used to stain for C7 in the cell medium. **(d)** Full Western Blot of WT, untreated and treated fibroblasts, Ponceau S used to stain for total protein in the cell medium as loading control.
